# Dopamine Enhances Healthspan and Locomotor Performance via Antioxidant Defense in Silkworms, *Bombyx mori*

**DOI:** 10.3390/insects17020205

**Published:** 2026-02-14

**Authors:** Songzhen He, Wenhao Yang, Hai Hu, Fangyin Dai, Xiaoling Tong

**Affiliations:** State Key Laboratory of Resource Insects, and Key Laboratory of Sericultural Biology and Genetic Breeding (Ministry of Agriculture and Rural Affairs), College of Sericulture, Textile and Biomass Sciences, Southwest University, Chongqing 400715, China; szhe@swu.edu.cn (S.H.); ygreatwriterh@163.com (W.Y.); huhaiswu@163.com (H.H.); fydai@swu.edu.cn (F.D.)

**Keywords:** silkworm moth, dopamine, lifespan, locomotor activity, antioxidant defense

## Abstract

The impact of dopamine on aging is complex and context-dependent. This study systematically investigated the effects of dopamine levels on the lifespan and behavior of the silkworm moth by employing genetic mutants of the dopamine synthesis/metabolic pathway combined with pharmacological interventions. The results demonstrated a significant positive correlation between endogenous dopamine levels and adult lifespan. Reducing dopamine levels shortened lifespan, whereas a moderate peripheral administration significantly extended healthspan, improved locomotor activity, and restored motor vigor in aged moths, though an excessive dose was toxic. The lifespan-extending effect of dopamine was closely correlated with enhanced total antioxidant capacity, observed in both long-lived strains and dopamine-treated individuals. Dopamine’s effects also exhibited sexual dimorphism and age dependency. This research reveals that a moderate increase in systemic endogenous dopamine or peripheral dopamine administration exerts positive effects on lifespan and aging-related behaviors, an effect likely mediated by enhanced antioxidant defense. These findings not only advance our understanding of the role of dopamine in the aging process but also provide a novel theoretical framework for developing anti-aging therapies and breeding superior silkworm strains.

## Simple Summary

As a key chemical messenger, the physiological roles of dopamine have been widely studied within the brain, but its precise effects on the aging process remain incompletely understood and lack a clear consensus. Here, we investigated the relationship between dopamine levels and both healthspan- and courtship-associated locomotor activity in the silkworm moth (*Bombyx mori*), an ideal model for aging research. By analyzing different silkworm strains with variations in endogenous dopamine levels in combination with pharmacological interventions, we showed that increasing dopamine enhanced the total antioxidant capacity, delayed aging, and improved locomotor activity. Importantly, we found that moderate administration in peripheral tissues (i.e., outside the brain) was enough to achieve these benefits and to reverse aging-related behavioral decline. Collectively, our work demonstrates that dopamine can modulate aging via pathways independent of the central nervous system, suggesting the potential for a novel therapeutic strategy to delay aging and providing a theoretical foundation for breeding more robust silkworms in sericulture via manipulation of dopaminergic pathways.

## 1. Introduction

Dopamine, a crucial neurotransmitter [1], also serves as an essential precursor in melanin synthesis [2,3]. Dopamine biosynthesis in insects begins with tyrosine, which is sequentially converted by tyrosine hydroxylase (encoded by the *Th* gene) and dopa decarboxylase [4]. The resulting dopamine is utilized for melanin production by enzymes like the Yellow protein (encoded by the *yellow* gene) and phenol oxidases (e.g., laccase 2) [4,5], as well as for cuticular sclerotization via enzymes such as N-acetyltransferase (encoded by the *iAANAT* gene) or the Ebony protein [3]. Melanism, resulting from melanin accumulation, is one of the most common forms of pigmentation [3,6,7,8,9]. Notably, insect melanism is often accompanied by pleiotropic physiological alterations, exerting broad effects on pathogen resistance, behavior, locomotor activity, developmental timing, and aging [10,11,12]. Studies indicate that the melanin precursor dopamine is involved in regulating aging [13,14]. Therefore, the multifunctional nature of dopamine suggests it may serve as a link connecting pigmentation with aging [11]. However, the precise mechanistic role of dopamine in regulating aging remains incompletely understood, and existing evidence presents significant contradictions.

The role of dopamine in aging is complex and appears to be influenced by critical experimental variables, leading to seemingly contradictory findings across studies. Evidence supporting a beneficial role comes from specific contexts: for instance, genetic variation in the dopamine synthesis enzyme dopa decarboxylase correlates with longevity in *Drosophila* [13]; enhancing dopaminergic transmission is necessary for, but not sufficient to, extending lifespan and sustaining function in aged flies [14], and pharmacologically elevating dopamine levels in the brain can extend lifespan in rodents [15,16]. Conversely, other contexts yield neutral or detrimental outcomes: reducing dopamine biosynthesis in the fly brains or in worms shows no effect on lifespan [17,18], whereas chronic L-DOPA treatment, which systemically elevates dopamine, can induce neurotoxicity and increase mortality [19,20]. We propose that these discrepancies do not merely reflect random noise but rather point to a mechanism dependent on both dosage and specific tissue compartment. The nature of the manipulation (genetic vs. pharmacological), the dosage or level of alteration, and the tissue specificity of the intervention are likely key determinants of the outcome. Therefore, a critical step forward is to systematically investigate these variables.

As a lepidopteran model, the silkworm presents several unique advantages in aging studies. First, silkworms are highly sensitive to exogenous substances, whether delivered by injection or orally [21]. Second, the absence of feeding in adults after eclosion eliminates confounding variables from nutritional intake. Third, various mutants for key enzymes in the dopamine synthesis/metabolic pathway are available [5,8,22,23,24,25], among others. These attributes facilitate the investigation of the role of dopamine in aging.

Previously, we found that melanic silkworm moths exhibited significantly extended lifespan and sustained viability during aging [10]. In this study, we aimed to investigate the underlying mechanism. Using silkworm as a model and employing mutant strains in the dopamine pathway, we determined the close association between the pleiotropic features of melanism and dopamine levels. Our study demonstrates that a moderate increase in systemic dopamine or peripheral dopamine administration significantly extends the lifespan and enhances locomotor activity in silkworm moths. These effects are correlated with a concomitant elevation in total antioxidant capacity.

## 2. Materials and Methods

### 2.1. Silkworm Strains

The experimental silkworm strains included the wild-type Dazao, its near-isogenic derivatives (Dz-*mln*, Dz-*Bm*, Dz-*ch*), and the *sch* mutant, all sourced from the Silkworm Gene Bank at Southwest University. All silkworms were reared on fresh mulberry leaves in an artificial climate chamber maintained at 25 °C and 75% relative humidity with a 12 h/12 h light/dark cycle.

### 2.2. Adult Lifespan Assay

For adult lifespan assay, the survival status of unmated moths was checked at intervals of no more than 3 h, and the time of death was recorded for each moth. Individuals were housed separately (one moth per separate small compartment) in an artificial climate chamber. The allocation of samples to the various compartments was randomized to control for positional bias. The chamber provided controlled conditions (25 °C, 75% relative humidity, 12 h/12 h light/dark cycle).

### 2.3. Detection of Dopamine Content

Silkworm moths from various experimental strains at 1-day and 7-day post-eclosion were preserved in pre-chilled 1.5 mL RNase-free centrifuge tubes containing hydrochloric acid buffer (composed of 122 mL/L concentrated hydrochloric acid and 881 mg/L L-ascorbic acid, adjusted to pH 3). The tubes were wrapped in aluminum foil for light protection and immediately stored at −80 °C. Three biological replicates were included per group. Homogenization was performed using a high-throughput tissue grinder (SCIENTZ-48, Ningbo Scientz Biotechnology Co., Ltd., Ningbo, China) with a pre-cooled module under light-protected conditions. The grinding process was conducted at 70 Hz for 2 min.

The ground whole-body homogenate was centrifuged at 4 °C and 13,800× *g* for 10 min. The supernatant was transferred to new foil-wrapped RNase-free tubes, boiled for 10 min to denature proteins, and cooled to room temperature. Subsequently, an equal volume of chloroform was added, followed by vortexing at 4 °C for 10 min and centrifuging at 4 °C (13,800× *g*, 10 min). The final supernatant was analyzed by ultra-performance liquid chromatography (UPLC, LC-30A, Shimadzu, Kyoto, Japan). Separation was achieved using a Shim-pack XR-ODS III column (2.0 mm × 75 mm, 1.6 μm, Shimadzu, Kyoto, Japan). The mobile phase consisted of 0.1 M citrate phosphate buffer (pH 2.68) containing 0.1 mM EDTA (Sangon Biotech (Shanghai) Co., Ltd., Shanghai, China), 0.3 mM sodium octanesulfonate (Sigma, St Louis, MO, USA), and 10% methanol (Sigma, St Louis, MO, USA). The flow rate was set at 0.2 mL/min, the injection volume was 5 μL, and the column oven temperature was maintained at 40 °C. Dopamine concentrations are expressed as micrograms per gram of tissue (μg/g tissue), normalized to tissue weight.

### 2.4. Drug Injection and Survival Time Assessment

All pharmacological agents were freshly prepared immediately before use. Dopamine hydrochloride was dissolved in saline to achieve concentration gradients of 0.5 M, 0.25 M, 0.125 M, and 0.005 M. Solutions of 3-iodotyrosine (a dopamine synthesis inhibitor) and β-alanine (a dopamine-depleting agent) were prepared at concentrations of 0.25 M and 0.125 M. All solutions were protected from light and stored on ice until use.

A volume of 10 μL of the corresponding drug solution was injected subcutaneously in the abdominal segment of newly emerged adult silkworm moths using a self-made injection device. The control group received an equal volume (10 μL) of saline. The dosage was chosen based on established effective ranges for insects [25,26] and validated in our pilot studies as optimal to induce the target physiological response while avoiding mortality or significant adverse effects.

Following injection, all moths were immediately transferred to an artificial climate chamber, with each moth kept individually in a separate small compartment. The chamber conditions were maintained constant at a temperature of 25 °C and a relative humidity of 75%. The time of death was systematically recorded every 3 h at most.

### 2.5. Measurement of Locomotor Velocity

The locomotor velocity of male moths was measured using an insect olfactory behavior assay system (Model SIM-4, Beijing Kasuo Zhonghe Technology Co., Ltd., Beijing, China). All assays were conducted under a constant ambient temperature of 25 °C. After system activation, the air pump was turned on until the airflow stabilized. Then, three female silkworm moths were placed in an odor source bottle to serve as a pheromone source. A single male moth, the test subject, was positioned at the center of the sample chamber. The time required for the male moth to move 5 cm toward the female odor source was recorded. The locomotor velocity (V) for each moth was calculated using the formula, V = D/T, where D is the fixed distance of 5 cm, and T is the time (in seconds) recorded for the moth to traverse that distance.

Locomotor activity data were analyzed using the GraphPad Prism 8. The velocity for each individual moth was calculated and used as an independent data point. Statistical comparisons between the control and treatment groups were performed using an ordinary one-way ANOVA followed by Dunnett’s multiple comparisons test, with n = 30 biological replicates (individual moths).

### 2.6. Determination of Total Antioxidant Activity

The total antioxidant capacity reflects the comprehensive activity level of all antioxidant substances within a sample. The sample preparation procedure was as follows: Silkworm moth tissue was rinsed twice with ice-cold phosphate-buffered saline (PBS) to remove residual impurities. Subsequently, the tissue was rapidly frozen using liquid nitrogen and ground into a fine powder. The powder was transferred into RNase-free 1.5 mL centrifuge tubes, rapidly frozen. The total antioxidant activity was determined strictly according to the manufacturer’s instructions of the assay kit (Suzhou Keming Biotechnology Co., Ltd., Suzhou, China).

## 3. Results

### 3.1. Dopamine Levels, Rather than Melanism, Mediate Lifespan Extension in Silkworm Moths

In the silkworm sex-controlled melanism (*sml*) mutant, we observed that melanic male moths live longer and are more active than wild-type males [10]. Remarkably, their lifespan also exceeds that of the non-melanic females within the same *sml* strain [10]. This finding contradicts the prevalent pattern of longer female lifespan in animals [27,28]. Therefore, we hypothesized that the melanic phenotype confers physiological traits that delay aging and extend lifespan.

**Figure 1:**
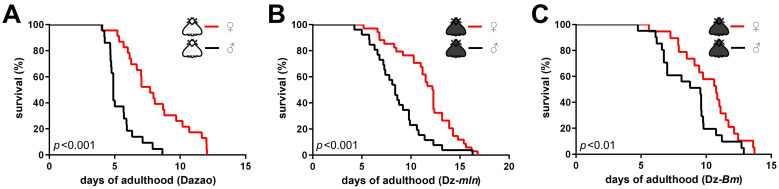
Unmated female moths outlive males within the same silkworm strain. Survival curves comparing unmated females and males. (**A**) In the Dazao strain, females (n = 46) lived significantly longer than males (n = 43). (**B**) In the Dz-*mln* strain, females (n = 34) lived significantly longer than males (n = 26). (**C**) In the Dz-*Bm* strain, females (n = 38) lived significantly longer than males (n = 41). (Log-rank test; *p* < 0.05 considered significant).

**Figure 2:**
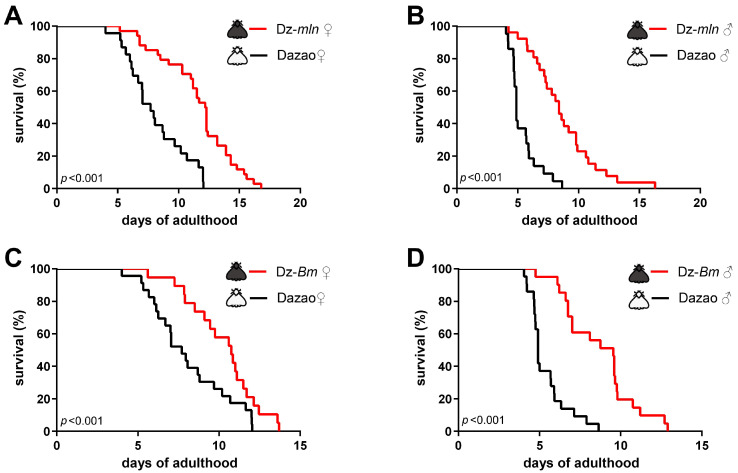
Melanic silkworm strains exhibit extended lifespan compared to the wild-type Dazao strain. Survival curves of unmated moths, comparing different strains within the same sex. (**A**,**C**) In females, both the Dz-*mln* (n = 34) and Dz-*Bm* (n = 38) strains lived significantly longer than the Dazao strain (n = 46). (**B**,**D**) In males, the Dz-*mln* (n = 26) and Dz-*Bm* (n = 41) strains also showed significantly extended lifespan compared to Dazao males (n = 43). (Log-rank test; *p* < 0.05 considered significant).

To validate our hypothesis, while minimizing the impact of genetic backgrounds, we first compared the lifespans of the wild-type Dazao strain and its near-isogenic melanic mutants, namely melanism (Dz-*mln*) and Black moth (Dz-*Bm*). The Dz-*mln* and Dz-*Bm* silkworm strains both exhibit melanic (black) moths in both sexes, whereas the wild-type Dazao strain is characterized by non-melanic (white) moths. Within the same strain, the lifespan of female moths was significantly longer than that of their male counterparts (Figure 1A–C), which aligned with the prevalent pattern [27]. Interestingly, within the same sex, the lifespans of both the black moth strains Dz-*mln* and Dz-*Bm* were significantly longer than that of the wild-type white moth Dazao (Figure 2). The result suggested a positive correlation between the melanic phenotype and delayed aging and extended lifespan in silkworm moths, thereby seemingly supporting our hypothesis.

**Figure 3:**
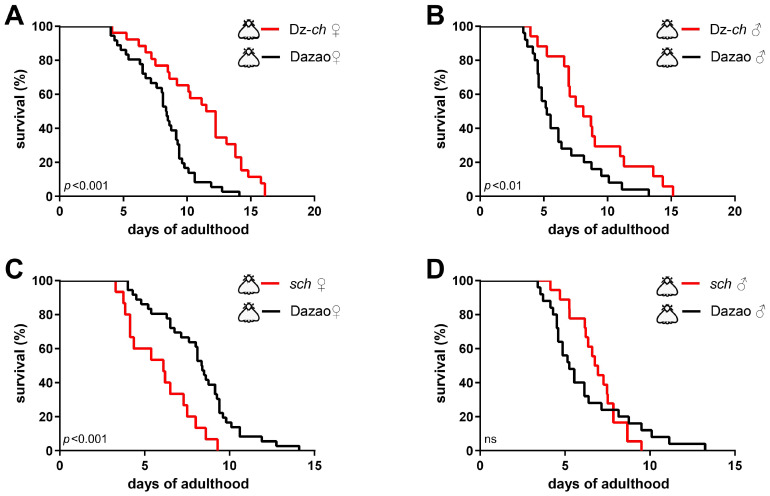
Non-melanic mutations in the melanin synthesis pathway exhibit divergent trends in adult lifespan. Survival curves in unmated, non-melanic mutants and wild-type (Dazao) controls. (**A**,**C**) In females, Dz-*ch* (n = 26) lived significantly longer, while *sch* (n = 30) lived significantly shorter than Dazao females (n = 36). (**B**,**D**) In males, Dz-*ch* (n = 34) also exhibited a significantly extended lifespan compared to Dazao (n = 25). Although the survival curves of *sch* (n = 36) and Dazao were not statistically different, the maximum lifespan was notably reduced in the *sch* strain. (Log-rank test; *p* < 0.05 considered significant; ns, not significant).

Since the melanic phenotype arises from melanin synthesis, to further probe the role of the dopamine/melanin pathway, we extended our analysis to two additional key mutants, namely chocolate (Dz-*ch*, *yellow* mutant) and sex-linked chocolate (*sch*, *Th* mutant). These strains exhibit a non-melanic (white) moth phenotype. To this end, we found that both males and females of the Dz-*ch* strain lived significantly longer than the wild-type Dazao (Figure 3A,B). Conversely, females of the *sch* strain had a significantly shorter lifespan than Dazao females (Figure 3C), and *sch* males showed a substantially reduced maximum lifespan (Figure 3D). Notably, the Dz-*ch* strain, despite its white color, achieved a lifespan comparable to the melanic strains (cf. Figure 2 and Figure 3A,B), indicating that longevity is not inextricably linked to the melanic phenotype.

Critically, the observed lifespan differences across these silkworm strains were consistent with dopamine levels predicted from their genetic backgrounds: the *sch* strain, characterized by reduced dopamine synthesis [24], shows a short lifespan (Figure 3C); while the Dz-*mln* (*iAANAT* mutant) [8,25] and Dz-*ch* [5] strains, which potentially accumulate dopamine, are long-lived (Figure 2A,B and Figure 3A,B). This preliminary pattern hinted at a potential positive correlation between endogenous dopamine levels and adult lifespan in silkworms.

**Figure 4:**
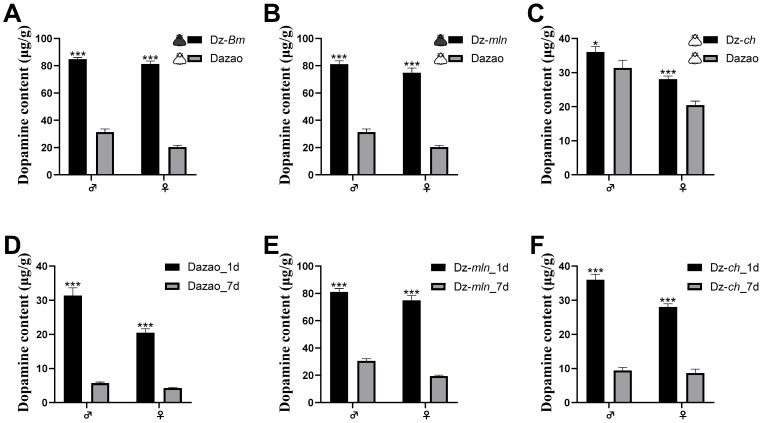
Dopamine levels are higher in long-lived strains and decline with aging. (**A**–**C**) Dopamine content in 1-day-old adults of long-lived strains (Dz-*Bm* (**A**), Dz-*mln* (**B**), and Dz-*ch* (**C**)) is significantly higher than in wild-type Dazao adults of the corresponding sex. (**D**–**F**) Longitudinal analysis of dopamine content shows a significant decrease from day 1 to day 7 post-eclosion in both sexes of the Dazao (**D**), Dz-*mln* (**E**), and Dz-*ch* (**F**) strains. Data are presented as mean ± SD (n = 3; Student’s *t*-test, * *p* < 0.05, *** *p* < 0.001).

To verify the correlation between systemic dopamine levels and the lifespan of silkworm moths, we further measured the dopamine content in multiple silkworm strains. Consistent with previous reports and our initial inference, adults of the long-lived strains—including the melanic Dz-*Bm* and Dz-*mln*, as well as the white moth strain Dz-*ch*—all exhibited significantly higher dopamine levels compared to the wild-type Dazao adults (Figure 4A–C). These findings demonstrate the positive correlation between the lifespan of silkworm moths and their dopamine content, suggesting that elevated dopamine levels, potentially as one of several factors due to its pleiotropic nature, may contribute to the longevity phenotype.

We further conducted a comparative analysis of dopamine levels in silkworm moths from the Dazao, Dz-*mln*, and Dz-*ch* strains at 1 and 7 days post-eclosion. The results showed a significant decrease in dopamine levels in both sexes at day 7 (senescence period) compared to day 1 (Figure 4D–F), demonstrating a marked decline in silkworm moth dopamine levels with aging, in agreement with extensive observations across species [29,30].

### 3.2. Reducing Dopamine Levels Shortens Lifespan in Silkworm Moths

**Figure 5:**
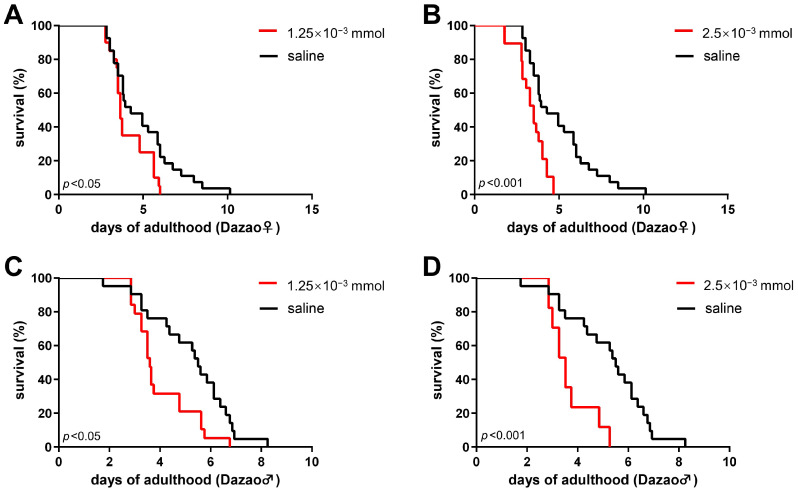
3-Iodotyrosine reduces silkworm lifespan in a dose-dependent manner. Survival curves of the Dazao strain following injection with 3-iodotyrosine or saline (control) on day 1 of adulthood. (**A**,**B**) In females, administration of 3-iodotyrosine at both low (1.25 × 10^−3^ mmol; n = 20) and high (2.5 × 10^−3^ mmol; n = 19) doses significantly reduced lifespan compared to the control group (n = 27). (**C**,**D**) In males, 3-Iodotyrosine also significantly reduced lifespan at both the low (n = 19) and high (n = 17) doses compared to the control (n = 21). The lifespan-shortening effect was greater at the high dose than at the low dose in both sexes (Log-rank test, *p* < 0.05 for low dose vs. control, *p* < 0.001 for high dose vs. control).

To further verify the relationship between dopamine and the adult lifespan of silkworm, we analyzed the impact of 3-iodotyrosine injection on their longevity. 3-Iodotyrosine, an inhibitor of tyrosine hydroxylase, results in a distinct reduction in dopamine in both the central nervous system (CNS) and peripheral tissues [31]. The results indicate that administering 3-iodotyrosine at doses of 2.5 × 10^−3^ mmol and 1.25 × 10^−3^ mmol significantly shortened the lifespan of both male and female silkworm moths (Figure 5), with a more pronounced effect observed at higher concentrations (Figure 5B,D).

**Figure 6:**
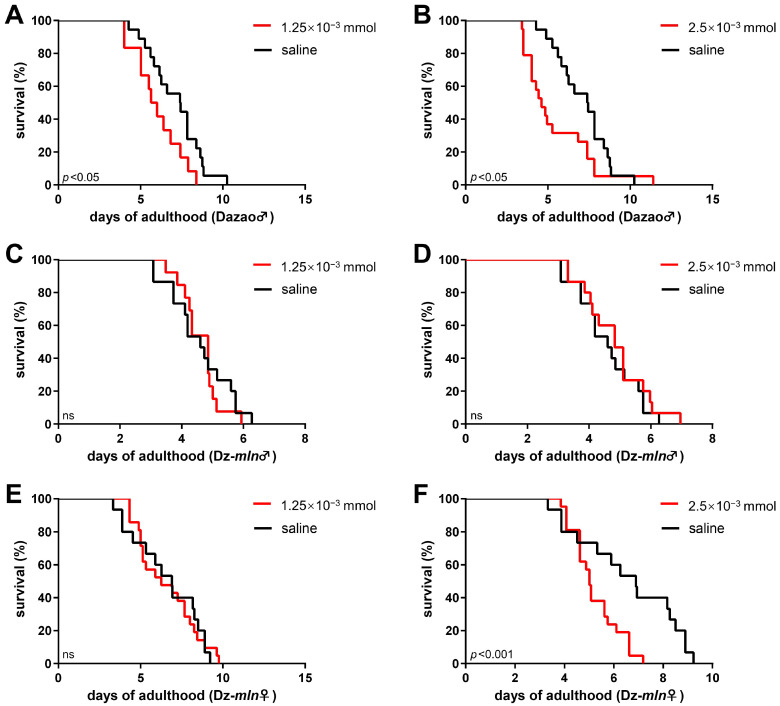
β-alanine injection shortens silkworm lifespan in a strain- and dose-dependent manner. Survival curves of the Dazao (wild-type) and Dz-*mln* (melanic mutant) strains after injection with β-alanine or saline (control) on day 1 of adulthood. (**A**,**B**) In the wild-type Dazao strain, administration of β-alanine significantly reduced lifespan at both low (1.25 × 10^−3^ mmol; n = 24) and high (2.5 × 10^−3^ mmol; n = 19) doses compared to the control (n = 18). (**C**,**D**) In Dz-*mln* males, the lifespan-shortening effect was attenuated: neither low (n = 26) nor high (n = 30) dose β-alanine significantly shortened lifespan relative to the control (n = 30). (**E**,**F**) In Dz-*mln* females, a significant reduction in lifespan was observed at the high dose (n = 21) but not at the low dose (n = 21) compared to the control (n = 30). (Log-rank test; *p* < 0.05 considered significant; ns, not significant).

We next investigated the effect of β-alanine injection on silkworm adult lifespan. β-Alanine triggers dopamine consumption [25,32,33]. In the wild-type Dazao strain, administration of β-alanine at both doses (1.25 × 10^−3^ mmol and 2.5 × 10^−3^ mmol) resulted in a significant reduction in lifespan (Figure 6A,B). By contrast, in the melanic mutant Dz-*mln* strain—characterized by elevated basal dopamine levels (Figure 4B)—a significant lifespan reduction was observed only at the high dose (Figure 6F), suggesting that depleting the larger dopamine pool in the Dz-*mln* mutant requires a stronger intervention. Furthermore, this high-dose effect was sexually dimorphic within the Dz-*mln* strain, significantly reducing lifespan in females but not in males (Figure 6D,F), indicating the greater sensitivity of females to this treatment.

Taken together, these results demonstrate that reducing dopamine levels—either by inhibiting its synthesis with 3-iodotyrosine or by promoting its consumption with β-alanine—consistently shortens the lifespan of silkworm moths.

### 3.3. Peripherally Administered Moderate Dopamine Extends Lifespan in Silkworm Moths

Research on dopamine in aging has predominantly focused on systemic or CNS-specific manipulations [13,14,15,16,17,18,19,20,34]; however, the precise role of dopamine remains controversial due to conflicting evidence. To specifically investigate the effects of peripheral administration on the adult lifespan of silkworms, we administered abdominal subcutaneous injections of dopamine hydrochloride at varying doses to adults of the Dazao strain on day 1 post-eclosion. This method leveraged the blood–brain barrier to confine the intervention to the peripheral system [35], thus enabling a specific elevation in peripheral dopamine levels.

**Figure 7:**
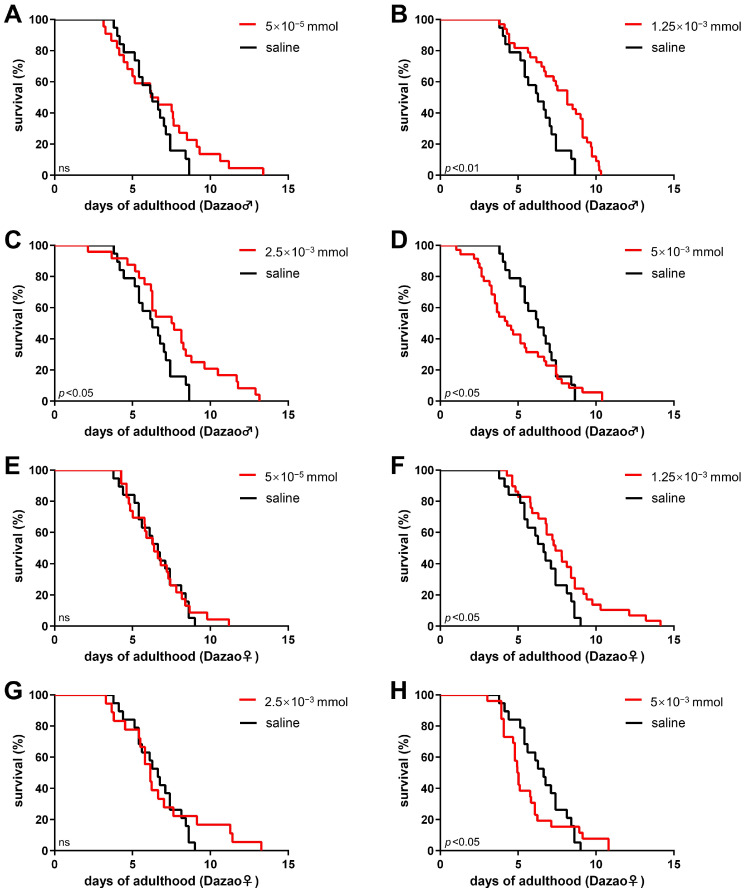
Dopamine exerts a biphasic, dose-dependent effect on silkworm lifespan. Survival curves of Dazao strain after injection with dopamine hydrochloride or saline (control) on day 1 of adulthood. (**A**–**D**) In males, dopamine administration resulted in a biphasic effect. (**A**) A low dose (5 × 10^−5^ mmol; n = 22) extended the maximum lifespan without a significant effect on overall survival curve (Log-rank test and Gehan–Breslow–Wilcoxon test). (**B**,**C**) Medium doses (1.25 × 10^−3^ mmol, n = 33; 2.5 × 10^−3^ mmol, n = 24) significantly prolonged lifespan (Log-rank test). (**D**) A high dose (5 × 10^−3^ mmol; n = 35) significantly shortened lifespan compared to the control (n = 19; Gehan–Breslow–Wilcoxon test). (**E**–**H**) In females, the effect was also dose-dependent. (**E**) The low dose (5 × 10^−5^ mmol; n = 21) had no significant effect (Log-rank test and Gehan–Breslow–Wilcoxon test). (**F**) A medium dose (1.25 × 10^−3^ mmol; n = 29) significantly prolonged lifespan (Log-rank test). (**G**) A medium–high dose (2.5 × 10^−3^ mmol; n = 18) extended the maximum lifespan but not the overall lifespan (Log-rank test and Gehan–Breslow–Wilcoxon test). (**H**) The high dose (5 × 10^−3^ mmol; n = 26) significantly reduced longevity relative to the control (n = 19; Gehan–Breslow–Wilcoxon test). (*p* < 0.05 considered significant; ns, not significant).

In male moths, a low-dose injection (5 × 10^−5^ mmol) of dopamine hydrochloride extended the population’s maximum lifespan, but had no significant effect on the overall lifespan (Figure 7A). However, at increased doses of 1.25 × 10^−3^ mmol and 2.5 × 10^−3^ mmol, a significant prolongation of the male moth lifespan was observed (Figure 7B,C). In contrast, the highest tested dose (5 × 10^−3^ mmol) significantly shortened the lifespan (Figure 7D). A similar trend was observed in female moths. The low-dose (5 × 10^−5^ mmol) treatment did not significantly alter lifespan (Figure 7E). The administration of a medium dose (1.25 × 10^−3^ mmol) significantly prolonged it (Figure 7F). The medium–high dose (2.5 × 10^−3^ mmol) extended the maximum lifespan, albeit without a significant increase in the overall lifespan (Figure 7G). Consistent with the results in males, the high dose (5 × 10^−3^ mmol) also significantly reduced female longevity (Figure 7H).

In conclusion, our findings demonstrate that dopamine hydrochloride exerts a typical dose-dependent biphasic effect on the adult lifespan of silkworms. Although subtle differences in the response to dosage existed between sexes, the overall trend was consistent: intermediate to medium–high doses (1.25 × 10^−3^ mmol to 2.5 × 10^−3^ mmol) significantly extended lifespan (Figure 7B,C,F), whereas the high dose (5 × 10^−3^ mmol) exhibited toxic effects, leading to lifespan shortening (Figure 7D,H). These findings indicate that moderate peripheral dopamine administration can effectively extend silkworm adult lifespan, although excessive elevation is counterproductive.

### 3.4. Dopamine Elevation Enhances Male Silkworm Moth Locomotor Activity

Being an important neurotransmitter, dopamine regulates diverse behaviors in insects, including olfactory and gustatory responses, learning and memory, as well as courtship and locomotor activity [36,37]. As we observed, enhanced locomotor activity was evident in both the melanic strains and dopamine-supplemented silkworm moths during rearing and experiments. To clarify the specific effects of dopamine on silkworm moth behavior, we evaluated the impact of exogenous dopamine treatment on male moth locomotor activity and compared the behavioral performances of silkworm strains with varying endogenous dopamine levels.

**Figure 8:**
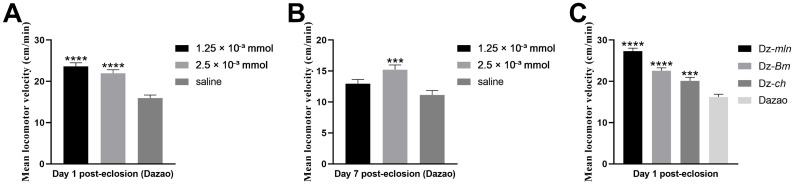
Dopamine enhances locomotor performance in silkworm moths. (**A**) The effect of exogenous dopamine hydrochloride injection on the locomotor velocity in 1-day-old male moths was quantified by an insect olfactory behavior assay system. Both medium (1.25 × 10^−3^ mmol) and high (2.5 × 10^−3^ mmol) doses significantly increased the velocity compared to the saline control. (**B**) Effect of dopamine injection on 7-day-old senescent male moths. The locomotor velocity was significantly enhanced by the high dose (2.5 × 10^−3^ mmol) but not by the medium dose (1.25 × 10^−3^ mmol). (**C**) Comparison of basal locomotor velocity among 1-day-old male moths of different strains with varying endogenous dopamine levels. The Dz-*mln*, Dz-*Bm* and Dz-*ch* strains, which have high endogenous dopamine, exhibited significantly higher velocity than the wild-type Dazao strain. Data are presented as mean ± SEM (n = 30; one-way ANOVA followed by Dunnett’s multiple comparisons test, *** *p* < 0.001, **** *p* < 0.0001).

We found that injecting dopamine hydrochloride (1.25 × 10^−3^ mmol or 2.5 × 10^−3^ mmol) to male moths on day 1 post-eclosion significantly enhanced their locomotor ability, manifested as an increased mean locomotor velocity as quantified by an insect olfactory behavior assay system (Figure 8A). In addition, when administered on day 7 post-eclosion (senescence period), the higher dose (2.5 × 10^−3^ mmol) restored locomotor velocity to a level comparable to young controls (cf. Figure 8A, saline), whereas the lower dose (1.25 × 10^−3^ mmol) produced no significant effect (Figure 8B). Moreover, one-day-old male moths from strains with higher endogenous dopamine levels (Dz-*mln*, Dz-*Bm*, Dz-*ch*) exhibited greater locomotor activity compared to wild-type Dazao males (Figure 8C). Therefore, these results demonstrate that dopamine modulates locomotor performance in silkworm moths, where a moderate increase in its levels significantly enhances locomotor activity.

### 3.5. Dopamine-Induced Healthspan Extension in Silkworm Moths Is Correlated with Enhanced Antioxidant Capacity

Given that the dopaminergic system is involved in the mediation of antioxidation in mammalian organisms [38], we hypothesized that the observed lifespan extension and improved locomotor performance in silkworm moths, resulting from a moderate elevation in dopamine levels, might also be associated with an enhancement of the organism’s overall antioxidant defense capacity. To test this hypothesis, we compared the total antioxidant capacity (T-AOC) among different silkworm strains with inherent variations in endogenous dopamine levels and examined the effects of exogenous dopamine administration on T-AOC.

**Figure 9:**
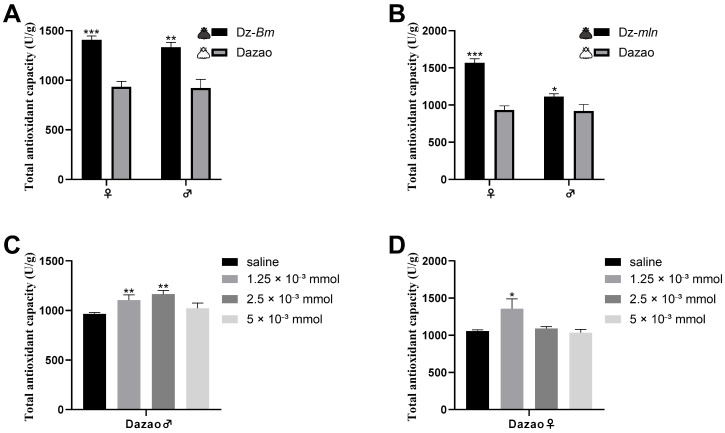
Association between dopamine levels and total antioxidant capacity on day 1 of adulthood. (**A**,**B**) Basal total antioxidant capacity (T-AOC) was significantly elevated in long-lived melanic strains (Dz-*Bm*, Dz-*mln*) with high endogenous dopamine compared to the wild-type Dazao strain. (**C**,**D**) Effect of exogenous dopamine hydrochloride injection on T-AOC in Dazao moths. (**C**) In males, T-AOC was significantly increased at doses of 1.25 × 10^−3^ mmol and 2.5 × 10^−3^ mmol compared to the saline control, but not at 5 × 10^−3^ mmol. (**D**) In females, a significant increase in T-AOC was observed at the 1.25 × 10^−3^ mmol dose, but not at higher doses. Data are presented as mean ± SD (n = 3; Student’s *t*-test, * *p* < 0.05, ** *p* < 0.01, *** *p* < 0.001).

Comparative analysis of one-day-old adults revealed that the long-lived, melanic strains (Dz-*Bm* and Dz-*mln*) exhibited significantly higher T-AOC levels than the wild-type Dazao strain in both sexes (Figure 9A,B). These results, combined with our previous findings of elevated overall endogenous dopamine levels in these melanic strains (Figure 4A,B), demonstrate a positive correlation between T-AOC and dopamine levels.

Exogenous dopamine treatment further confirmed this relationship. At a dose of 1.25 × 10^−3^ mmol, T-AOC was significantly increased in both male and female Dazao moths. At the increased dose of 2.5 × 10^−3^ mmol, a significant elevation in T-AOC was maintained in male moths, whereas this effect was not statistically significant in females. However, at the high dose of 5 × 10^−3^ mmol, T-AOC in both sexes showed no significant difference compared to the control group (Figure 9C,D). This dose-dependent effect aligns well with the lifespan-extending effect of moderate dopamine elevation that we observed previously (Figure 7), collectively indicating that moderate peripheral dopamine administration enhances antioxidant capacity and extends longevity, whereas an excessive dose produces no effect and may even induce detrimental effects.

In conclusion, our findings demonstrate that a moderate increase in overall endogenous dopamine or peripheral dopamine administration significantly extends silkworm moth lifespan and improves locomotor performance, an effect appearing to be mediated by the marked enhancement of the body’s total antioxidant capacity.

## 4. Discussion

Our study was initially inspired by the observed extended lifespan in the melanic moth of the *sml* mutant. By analyzing genetic mutants of the dopamine synthesis/metabolic pathway—which exhibit naturally varying endogenous dopamine levels—in combination with pharmacological interventions, we established that a moderate increase in dopamine significantly extends healthspan and enhances pheromone-directed male locomotor activity. This effect is likely mediated by an enhancement of the body’s total antioxidant capacity. Our prior transcriptomic analysis of the *sml* strain, which revealed a significant upregulation of the catalase gene [10], supports the proposal that this effect is mediated by an enhancement of total antioxidant capacity. Furthermore, in contrast to previous studies focusing primarily on CNS functions of dopamine [13,14,39,40], we found that peripheral administration of dopamine hydrochloride was sufficient to enhance antioxidant capacity, improve longevity and male moth locomotor performance, and restore motor vigor in aged moths, highlighting the significance of peripheral signaling in aging modulation.

A notable finding was the pronounced sexual dimorphism in silkworm moth sensitivity to dopamine fluctuations. In the Dz-*mln* mutant, treatment with β-alanine resulted in a significant reduction in lifespan specifically in females, with no significant change observed in males (Figure 6D,F), indicating that female individuals exhibit higher sensitivity to this treatment. We speculate that this disparity may be attributed to two non-mutually exclusive factors: first, the intrinsically lower basal dopamine levels in females (Figure 4B), which might render them more susceptible to further dopamine depletion; second, the previously documented greater plasticity in lifespan regulation among female silkworms [27]. Furthermore, differences in tolerance and response thresholds to exogenous dopamine were observed between sexes in the wild-type Dazao strain, with females exhibiting a lower threshold dose for adverse effects compared to males. Specifically, injection at a medium–high dose (2.5 × 10^−3^ mmol) significantly increased the T-AOC and extended the lifespan in males, while no significant effect was observed in females (Figure 7C,G and Figure 9C,D), suggesting that this dose may already exceed the optimal range for female lifespan extension. This consistent pattern across strains aligns with the known mechanism that sexual dimorphism in neurotransmitter systems (particularly the dopamine system) often leads to differential drug responses [41], suggesting that the greater sensitivity of female silkworm moths to dopamine fluctuations and pharmacological interventions may represent a conserved physiological trait.

In addition to sexual dimorphism, dopamine-induced improvements also show a marked age-dependent effect. In young silkworm moths (1-day post-eclosion), both lower (1.25 × 10^−3^ mmol) and higher (2.5 × 10^−3^ mmol) doses significantly enhanced the pheromone-directed locomotor ability of male moths, with the lower dose producing a more pronounced improvement (Figure 8A). In contrast, in senescent moths (7-day post-eclosion), the lower dose failed to significantly enhance locomotor activity, whereas the higher dose effectively restored their vigor to a level comparable to the young control group (Figure 8B; cf. Figure 8A, saline control). This age-dependent efficacy is likely due to an age-related decline in both basal dopamine levels (Figure 4D) and the dopaminergic function [29]. Therefore, by moderately elevating dopamine levels, locomotor function in aged moths was successfully restored to a youthful state, underscoring the promising therapeutic potential of this approach against age-related locomotor decline.

Our findings of a beneficial, antioxidant-mediated effect from moderate peripheral dopamine administration may appear to contrast with reports of neurotoxicity following chronic, systemic L-DOPA administration or the limited effects of brain-specific dopamine reduction. However, this discrepancy can be reconciled by key differences in dopamine dynamics tied to dosage and anatomical site of action. First, the neurotoxicity associated with chronic, high-dose systemic L-DOPA likely results from supra-physiological dopamine fluxes that promote oxidative stress [42,43], whereas our moderate peripheral administration enhances antioxidant capacity without overwhelming redox homeostasis. Second, peripheral dopamine signaling may bypass central toxicity mechanisms associated with L-DOPA metabolism [42]. Finally, and crucially, targeted reduction in dopamine in specific brain regions may fall below the threshold required to impact organismal aging under baseline conditions, or its effects may be compensated by other pathways, which could explain the limited effects observed in some studies. Collectively, our results demonstrate that the beneficial window for dopamine signaling is critically constrained by both dosage and tissue compartment of action.

Furthermore, it is important to note that direct extrapolation of these findings to other organisms should be considered with caution, as physiological and genetic differences between Lepidoptera and other taxa may influence how dopamine modulates aging. Nevertheless, the silkworm serves as a valuable model for revealing evolutionarily conserved mechanisms. We also note that the mechanistic insights from our current study, while robust, are based on a relatively small sample size. Future work should therefore involve larger cohorts across different species, coupled with direct measurement of tissue dopamine levels following pharmacological interventions, to strengthen the evidence for the proposed mechanism and to test its generalizability. To fully characterize healthspan enhancement, subsequent studies should also incorporate a wider array of age-related functional benchmarks beyond the pheromone-directed locomotion measured here.

In summary, this study elucidates dopamine’s role in extending silkworm moth lifespan and enhancing locomotor activity, establishes the critical importance of its peripheral signaling, and delineates the significant influence of sex and age on these effects. Our data support a model whereby the lifespan extension is primarily achieved via boosted antioxidant defense, while the locomotor behavior enhancement likely involves distinct receptor-mediated pathways. Given that adult silkworms allocate nearly all physiological resources to reproduction, our work on this economically important insect provides a rationale for potential molecular breeding strategies targeting dopamine-related pathways to develop more robust and reproductively vigorous strains. However, the direct application of these findings in sericulture requires validation under practical conditions, specifically through demonstrations of improved mating success, fecundity, and sperm competitiveness. Beyond these translational aims, a deeper mechanistic understanding is crucial. Therefore, building on these findings, a primary objective for future work is to delineate the specific molecular pathways—including the receptor subtypes and downstream cascades associated with antioxidant defense versus locomotor behavior—to clarify whether these effects share common mechanisms or operate through parallel pathways.

## Data Availability

The original contributions presented in this study are included in the article. Further inquiries can be directed to the corresponding author.
